# The hypoparathyroidism-associated mutation in *Drosophila* Gcm compromises protein stability and glial cell formation

**DOI:** 10.1038/srep39856

**Published:** 2017-01-04

**Authors:** Xiao Xi, Lu Lu, Chun-Chun Zhuge, Xuebing Chen, Yuanfen Zhai, Jingjing Cheng, Haian Mao, Chang-Ching Yang, Bertrand Chin-Ming Tan, Yi-Nan Lee, Cheng-Ting Chien, Margaret S. Ho

**Affiliations:** 1 Research Center for Translational Medicine, Shanghai East Hospital, Tongji University School of Medicine, No. 150 Jimo Road, Shanghai 200120, China; 2 Key Laboratory of Arrhythmias of the Ministry of Education of China, Shanghai East Hospital, Tongji University School of Medicine, No. 150 Jimo Road, Shanghai 200120, China; 3 Department of Anatomy and Neurobiology, 1239 Siping Road, Tongji University School of Medicine, Shanghai, 200092, China; 4 Department of Biomedical Sciences and Graduate Institute of Biomedical Sciences, College of Medicine, Chang Gung University, Kwei-San, Tao-Yuan, Taiwan; 5 Institute of Molecular Biology, Academia Sinica, Taipei, Taiwan

## Abstract

Differentiated neurons and glia are acquired from immature precursors via transcriptional controls exerted by factors such as proteins in the family of Glial Cells Missing (Gcm). Mammalian Gcm proteins mediate neural stem cell induction, placenta and parathyroid development, whereas *Drosophila* Gcm proteins act as a key switch to determine neuronal and glial cell fates and regulate hemocyte development. The present study reports a hypoparathyroidism-associated mutation R59L that alters *Drosophila* Gcm (Gcm) protein stability, rendering it unstable, and hyperubiquitinated via the ubiquitin-proteasome system (UPS). Gcm^R59L^ interacts with the Slimb-based SCF complex and Protein Kinase C (PKC), which possibly plays a role in its phosphorylation, hence altering ubiquitination. Additionally, R59L causes reduced Gcm protein levels in a manner independent of the PEST domain signaling protein turnover. Gcm^R59L^ proteins bind DNA, functionally activate transcription, and induce glial cells, yet at a less efficient level. Finally, overexpression of either wild-type human Gcmb (hGcmb) or hGcmb carrying the conserved hypoparathyroidism mutation only slightly affects gliogenesis, indicating differential regulatory mechanisms in human and flies. Taken together, these findings demonstrate the significance of this disease-associated mutation in controlling Gcm protein stability via UPS, hence advance our understanding on how glial formation is regulated.

A fundamental issue during development is how individual cells acquire their identities from undefined precursors and mature into distinct cell types with functional features. For instance, during the development of a nervous system, neurons and glia arise from common precursors and share similar origins. Neural precursors receive instructive cues upon proper signaling and are given a choice of differentiating into either neurons or glia. Initial precursor cell fate determination, and the key events that follow to regulate their differentiation, have always been subjects of high interest. In the past, transcription factors that regulate gene expression have been shown to play pivotal roles during these processes. By turning on downstream gene transcription, transcription factors activate signal transduction pathways that construct the overall transformation; they are hence extremely important targets for tight regulation on their activity.

In the animal organism *Drosophila melanogaster*, neural cell specification and differentiation share similarity with the mammalian system. *Drosophila* embryonic neural stem cells (NSCs), also named neuroblasts (NBs), are plastic with undefined nature and serves as an excellent model to study stem cell biology^1^,^2^,^3^. During embryonic neurogenesis, NBs undergo asymmetric division to generate a smaller ganglion mother cell (GMC), which divides once more to produce differentiated neurons and/or glial cells, and another NB with self-renewal potential^1^,^4^. Interestingly, the transcription factor *glial cell missing/glial cell deficient (gcm/glide*, henceforth *gcm*) has been shown to act as a binary switch in controlling neuronal and glial cell fates^5^,^6^,^7^,^8^,^9^,^10^. Not only does Gcm play a role in neural precursor cell specification, it also promotes glial differentiation by activating downstream target genes such as *reversed polarity (repo*) and *pointed (pnt*)^11^,^12^, and represses the neuronal fate through the activation of *tramtrack (ttk*)^13^,^14^. *gcm* and its homologous gene, *gcm2*, also play a role during hemocyte development^15^,^16^. Non-neuronal functions for mammalian counterparts have also been reported – whereas the mammalian *gcm* homolog *gcma* (also named *gcm1*) controls the differentiation of the syncytiotrophoblast layer during placenta development^17^,^18^, mutations of a *gcm* homolog *gcmb* (also named *gcm2*) has been found in patients carrying the hypoparathyroidism disease^19^,^20^,^21^. More recently, new studies have shown that genes in the mammalian *gcm* family are essential for neural stem cell induction, further strengthening the role of these proteins in the developing nervous system^22^,^23^. Together, these findings have underscored the importance of Gcm proteins and make them reasonable targets for precise regulation on their activity.

Previous studies have indicated that Gcm proteins exhibit differences in stability and are under tight regulation via post-translational modification^24^,^25^,^26^,^27^. Moreover, Gcm proteins have been shown to undergo regulated degradation via the ubiquitination-proteasome system (UPS), a widely used mechanism to control protein turnover^28^,^29^. The UPS degradation machinery comprises a major enzymatic cascade that targets and covalently links ubiquitin (Ub) chains to specific substrates. After the E1 activating enzyme utilizes ATP to form a high-energy thioester bond with Ub, the activated Ub is transferred to the E2 conjugating enzyme. The E3 ligase, either HECT or cullin-based RING type, recognizes specific substrates and catalyzes Ub-substrate conjugation from E2. Ultimately, the ubiquitinated substrates are sent for destruction by the 26 S proteasome. A number of E3 ligases have been identified. Among all, the S phase kinase-associated protein 1 (SKP1)–cullin 1 (CUL1)–F-box protein (SCF) complex, a better-studied multi-subunit E3 ligase, provides the substrate specificity via the adaptor F-box protein^30^,^31^. Substrates targeted for ubiquitination are often phosphorylated and interact with the substrate-binding domain of F-box protein (like WD repeats or leucine-rich repeats LRR).

Intriguingly, our previous studies and others have demonstrated that SCF complex mediates Gcm protein degradation and Gcm interacts with the F-box protein Supernumerary limbs (Slimb) and Archipelago (Ago)^28^. Furthermore, excessive or insufficient amount of Gcm, due to dysregulation on its UPS degradation, leads to a destructive imbalance that causes defective gliogenesis, demonstrating the necessity to precisely regulate Gcm protein stability^6^,^28^. Despite so, the detailed mechanisms of how Gcm is post-transcriptionally modified and accessed by the degradation machineries remain elusive. Here we present evidence that Gcm proteins carrying a hypoparathyroidism-related mutation R59L (Gcm^R59L^) are intrinsically destabilized and exhibit a shorter half-life due to altered phosphorylation and hyperubiquitination. Gcm^R59L^ proteins interact with the Slimb-based SCF complex and Protein Kinase C (PKC), which possibly plays a role in regulating Gcm^R59L^ phosphorylation. Gcm^R59L^ proteins also retain the ability to bind DNA and activate transcription. Furthermore, R59L mutation alters Gcm protein stability in a manner independently of the PEST domain implicated in rapid protein turnover. *In-vivo*, whereas Gcm^R59L^ proteins induce an increase in glial cell number similarly as the wild-type Gcm, this increase is not sustained due to the reduced protein level. In contrast, glial cell number is not largely affected when human Gcmb (hGcmb) carrying the same mutation is overexpressed. These results suggest that the disease-related R59L mutation causes distorted Gcm protein stability via SCF complex-dependent ubiquitination and degradation, hence playing an important role in glial cell development.

## Results

### N-terminal R59 renders Gcm unstable

**Figure 1:**
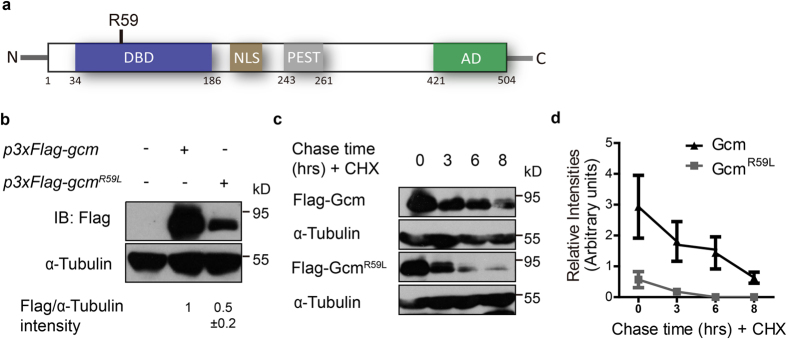
Gcm^R59L^ proteins are intrinsically unstable. (**a**) Protein structure of *Drosophila* Gcm. Gcm proteins contain a DNA binding domain (DBD, blue), a nuclear localization signal sequence (NLS, brown), a PEST domain (PEST, gray), and a transactivation domain (AD, green). R59 locates in the DNA binding domain (DBD). (**b**) Lysates from S2 cells transfected with *p3xFlag-gcm* or *p3xFlag-gcm*^*R59L*^ were harvested and analyzed by Western blot analysis. Quantifications for relative band intensity of Flag to α-Tubulin were shown at the bottom. Note that Gcm^R59L^ protein levels were about two-fold lower than Gcm. (**c**) Pulse chase analysis on S2 cells expressing Gcm or Gcm^R59L^ treated with CHX (10 μM). Lysates were collected at 0, 3, 6, and 8 h after CHX treatment. Control: α-Tubulin. (**d**) Quantifications for relative band intensities of Flag to α-Tubulin at each time point from 3 independent experiments (n = 3). Half-life for Gcm is about 5 h and for Gcm^R59L^ is about 1.5 h. Western blot gels have been run under the same experimental conditions. * represents p < 0.05, ** represents p < 0.01, and *** represents *p* < 0.001 by Student’s t test.

Our previous investigation has shown that Gcm undergoes ubiquitination-mediated protein degradation and its protein stability is crucial for glial cell development in *Drosophila* embryos^28^. To investigate the degradation mechanism in detail, point mutations in different regions of Gcm were created by PCR site-directed mutagenesis and tested for their impact on protein stability (Fig. S1). These mutations target conserved residues predicted to undergo post-translational modification or related to disease pathology and include: mutations in the DNA binding regions (C93S, C93A, C103A, C118A, and C144A), potential slimb-binding sites (S39A), potential sumolyation sites (R175A and K243A), and potential phosphorylation sites (T217A and T416A) (Fig. S1). Among all, N-terminal residue Arginine59 within the DNA binding domain was changed to Leucine (R59L), a change similar to a conserved mutation in the mammalian *gcmb* gene carried by hypoparathyroidism patients (Fig. 1a)^19^,^32^,^33^,^34^. To examine whether this mutation affects Gcm protein stability, cell lysates from *Drosophila* S2 cells transfected with Gcm or Gcm carrying the R59L mutation were harvested and analyzed by SDS-PAGE. These proteins were engineered with a Flag epitope tag in three tandem repeats (3xFlag) at the N-terminus (henceforth denoted as Gcm or Gcm^R59L^ in the text). Western blot and quantitative analyses showed that Gcm^R59L^ protein levels exhibited a significant two-fold decrease compared to Gcm, indicating that Gcm^R59L^ proteins are less stable and intrinsically destabilized due to the alternation on this particular residue R59 (Fig. 1b).

Next, half-lives of Gcm and Gcm^R59L^ proteins were examined. Consistent with our previous observation^27^, Gcm protein levels were reduced by two-fold about five hours after the addition of cycloheximide (CHX) (Fig. 1c,d). Interestingly, Gcm^R59L^ proteins exhibited a much shorter half-life of approximately 1.5 hours (Fig. 1c,d). Results from these pulse chase analyses indicated that Gcm^R59L^ proteins decay in a much faster rate compared to Gcm, and are potentially more accessible to the degradation machineries. To rule out the possibility that the reduction in protein levels was due to a change in the transcriptional level, equal amount of *gcm* or *gcm*^*R59L*^ DNA was used for transfection and their mRNA transcripts were examined using reverse transcription polymerase chain reaction (RT-PCR, Supplementary Fig. S2a). Our results showed no detectable difference in the levels of *gcm* or *gcm*^*R59L*^ mRNA transcripts, suggesting that the key step in regulating Gcm^R59L^ protein stability occurs at the post-transcriptional level.

### Gcm^R59L^ protein stability is controlled by ubiquitin-mediated degradation

**Figure 2:**
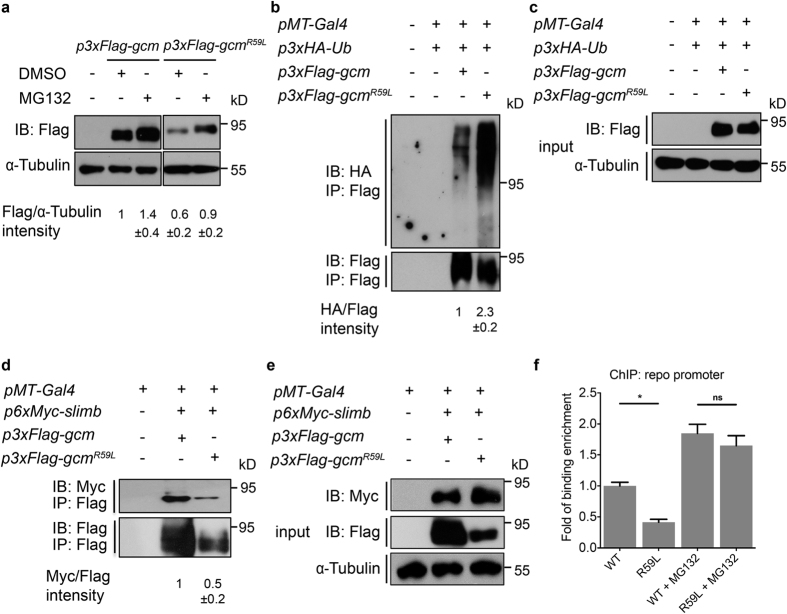
Gcm^R59L^ proteins are hyperubiquitinated. (**a**) Western blots showing expressions of Gcm and Gcm^R59L^ in S2 cells in the absence or presence of MG132 (50 μM). Lysates were collected at 4 h after MG132 treatment. Quantifications for relative band intensity of Flag to α-Tubulin were shown at the bottom. (**b**,**c**) Co-IP analysis of cells co-transfected with *p3xHA-Ub* and *p3xFlag-gcm* or *p3xFlag-gcm*^*R59L*^. Ubiquitinated Gcm and Gcm^R59L^ were detected as smears using anti-HA antibody after immunoprecipitation with anti-Flag antibody. Quantifications for relative band intensity of HA to Flag were shown at the bottom. Inputs were shown in (**c**). Note that a much higher ubiquitination level was detected for Gcm^R59L^. (**d**,**e**) Co-IP analysis of S2 cell lysates co-transfected with *p6xMyc-Slimb* and *p3xFlag-gcm* or *p3xFlag-gcm*^*R59L*^ indicated that both Gcm and Gcm^R59L^ bind Slimb. Quantifications for relative band intensity of Myc to Flag were shown in (**d**). Inputs were shown in (**e**). (**f**) Gcm^R59L^ binds DNA. ChIP assays were performed using S2 cell lysates transfected with *p3xFlag-gcm* or *p3xFlag-gcm*^*R59L*^ in absence or presence of MG132 (50 μM). For quantification, results were normalized against the level for Gcm without MG132 treatment (designated as 1.0). Each ChIP experiment was repeated three times (n = 3). Note a drop in the fold of binding enrichment when cells are transfected with *p3xFlag-gcm*^*R59L*^. No significant difference was detected for cells transfected with *p3xFlag-gcm* or *p3xFlag-gcm*^*R59L*^ in the presence of MG132. Averages are mean ± standard error of mean (SEM), *represents p < 0.05, **represents p < 0.01, and ***represents *p* < 0.001 by Student’s t test. ns means no significance. Western blot gels have been run under the same experimental conditions.

Since Gcm^R59L^ proteins are intrinsically destabilized, it is intriguing to speculate whether the reduction in Gcm^R59L^ protein levels is due to an altered efficiency of its ubiquitination and degradation. To test this idea, S2 cells transfected with Gcm or Gcm^R59L^ were first treated with the proteasome inhibitor MG132 to block the 26 S proteasome-dependent degradation. Both Gcm and Gcm^R59L^ proteins accumulated upon the treatment, suggesting that Gcm^R59L^ protein stability is also under 26 S proteasome control, albeit with a lower protein amount (Fig. 2a). On the other hand, we found that treatment with the lysosome inhibitor E64 did not greatly affect protein stability of either Gcm or Gcm^R59L^, suggesting that 26 S proteasome pathway is the major route for their degradation (Supplementary Fig. S2b,c).

Next, ubiquitination levels of Gcm proteins were analyzed by co-transfecting Gcm or Gcm^R59L^ with ubiquitin carrying a HA epitope tag (HA-Ub) in S2 cells. HA antibody was used to detect the ubiquitinated fractions in the pull-downs of Gcm or Gcm^R59L^ proteins (Fig. 2b,c). As expected, a lower amount of Gcm^R59L^ proteins were pulled down using the Flag antibody due to its lower protein levels. However, a strikingly high level of ubiquitination was detected among these Gcm^R59L^ proteins (Fig. 2b). Densitometric analysis revealed a two-fold increase in the normalized Gcm^R59L^ ubiquitination level (HA/Flag intensities) as compared to that of Gcm (Fig. 2b). Furthermore, given the established notion that the F-box protein Supernumerary limbs (Slimb) is the major substrate adaptor in mediating Gcm ubiquitination^28^, our analysis here showed that Gcm^R59L^ proteins interacted with Slimb, suggesting that Slimb may also act as a substrate adaptor for Gcm^R59L^ protein ubiquitination (Fig. 2d,e). *In toto*, these results indicate that the mutation R59L does not prevent Gcm binding to Slimb and the reduction in Gcm^R59L^ protein levels is likely due to hyperubiquitination.

### Gcm^R59L^ binds to DNA

Due to the fact that R59 is located within the DNA binding domain, it is possible that R59L mutation affects Gcm binding to DNA. To address this issue, the association between Gcm or Gcm^R59L^ and the endogenous promoter of Gcm target gene *reverse polarity (repo*) was analyzed using chromatin immunoprecipitation assays (ChIP). Initially, a two-fold reduction in the extent of promoter association by Gcm^R59L^ was detected compared to that of wild-type Gcm (Fig. 2f). The reduction in the protein-DNA interaction may be attributed to either a defect in the DNA binding or the lower Gcm^R59L^ protein level. To distinguish between these two possibilities, ChIP assays were performed again in the presence of MG132. Upon MG132 addition, there was no discernable difference in the chromatin occupancy of Gcm or Gcm^R59L^ at the endogenous *repo* promoter (Fig. 2f). By blocking the 26 S proteasome pathway, Gcm^R59L^ protein levels were preserved and an increase in the protein-DNA interaction was detected. These results thus suggest that Gcm^R59L^, albeit with a lower stability, likely exhibits similar DNA binding capacity as the wild-type Gcm.

### Gcm^R59L^ overexpression increases glial cell number

**Figure 3:**
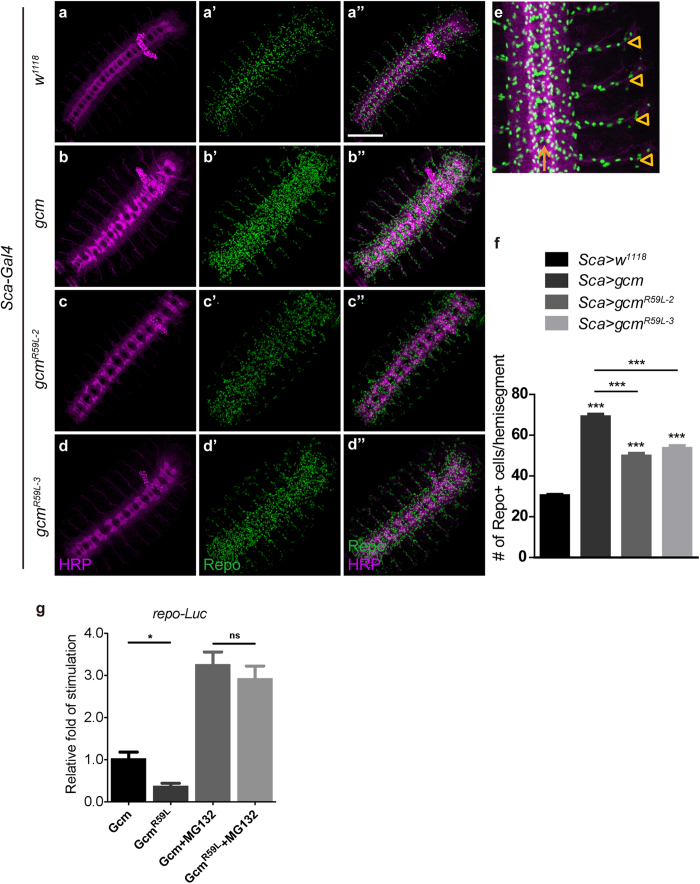
Gcm^R59L^ overexpression increases glial cell number less efficiently than Gcm. (**a**–**d”**) Confocal projections of Stage 15 embryos immunostained with HRP (magenta) to reveal neuronal membranes and Repo (green) for glia for the following genotypes: (**a**–**a”**) *Sca-Gal4* > *w*^*1118*^ (Control), (**b**–**b”**) *Sca-Gal4* > *UAS-gcm*, (**c**–**c”**) *Sca-Gal4* > *UAS-gcm*^*R59L-2*^, and (**d**–**d”**) *Sca-Gal4* > *UAS-gcm*^*R59L-3*^. Scale bar: 100 μm. (**e**) Numbers of Repo-positive cells per hemisegment were scored from the midline (yellow arrow) to the 5 LCH-associated glia (yellow arrowheads). (**f**) Numbers of glia per hemisegment in Stage 15 were shown for embryos with the above genotypes. Repo-positive cells were count for hemisegments A1–A4, with n = 24 (*w*^*1118*^), n = 48 (*UAS-gcm*), n = 30 (*UAS-gcm*^*R59L-2*^), and n = 40 (*UAS-gcm*^*R59L-3*^). (**g**) Luciferase reporter assays were performed for cells transfected with *p3xFlag-gcm* or *p3xFlag-gcm*^*R59L*^ in the absence or presence of MG132 (50 μM). Note that a decrease in the activity was seen for Gcm^R59L^. In the presence of MG132, however, no difference was detected for either form. Averages are mean ± SEM, * represents p < 0.05, ** represents p < 0.01, and *** represents *p* < 0.001 by Student’s t test between two groups and one-way ANOVA test among multiple groups. ns means no significance.

Our biochemical data suggest that Gcm^R59L^ proteins are much less stable compared to the wild-type Gcm, hence may be functionally compromised. We tested this idea by overexpressing Gcm or Gcm^R59L^ in neural precursors using *Scabrous-Gal4 (Sca-Gal4*). Previous results and ours have confirmed that Gcm overexpression in these cells results in a massive increase in glial cell population (Fig. 3b–b”). Gcm^R59L^ overexpression also caused glial cell induction, albeit to an apparently lesser extent (Fig. 3c–d”). Statistical analysis indicated that whereas Gcm overexpression in neural precursors has resulted in a roughly two-fold increase in glial cell number (30.2 ± 0.7 to 68.9 ± 1.3 cells per hemisegment, p < 0.001, Fig. 3f), the effect of Gcm^R59L^ overexpression was not as significant (30.2 ± 0.7 to 49.7 ± 1.4 or 53.4 ± 1.4 cells per hemisegment, p < 0.001, Fig. 3f). Number of glial cells was counted according to previous protocols for regions from the midline (yellow arrow) to the five lateral chordotonal (LCH) organ-associated glia (yellow arrowheads) (Fig. 3e)^28^. Similar effects were seen when two copies of Gcm or Gcm^R59L^ transgenes were overexpressed in neural precursors (Supplementary Fig. S3). To rule out the positional effect of different transgene insertions, two independent transgenic fly lines carrying *UAS-gcm*^*R59L*^ were analyzed. Similar results were obtained for both lines (Fig. 3c–d”). Taken together, these results indicate that Gcm^R59L^ also possesses the ability to induce glial cell formation; yet by carrying this point mutation, it does not work as efficiently as the wild-type Gcm.

### Gcm^R59L^ functionally activates gene transcription

Our *in-vivo* analysis on glial cell formation has suggested that Gcm^R59L^ functions in a less competent manner. We then sought to determine whether the incompetency is due to a weakened ability to activate transcription. To analyze Gcm transcriptional activity, luciferase reporter assays were designed and performed to monitor gene expression from the Gcm downstream target *repo* promoter in S2 cells. As shown in Fig. 3g, a significant decrease in transcriptional activity represented by the fold of stimulation was detected for Gcm^R59L^. Yet, treatment with MG132 greatly enhanced the activity, suggesting that, as in the case of chromatin DNA binding, Gcm^R59L^ proteins work as efficiently as the wild-type Gcm to activate gene transcription (Fig. 3g). These lines of evidence suggest that Gcm^R59L^ functionally activates gene transcription and that the less productive transactivation activity could be attributed to its intrinsic loss of stability.

### Gcm^R59L^ is less competent in glial cell induction

Results from Gcm and Gcm^R59L^ overexpression suggest that glial cell formation is functionally induced, yet different number of Repo-positive cells were detected when stage 15 embryos were analyzed. We then sought to determine if the attenuated Repo-positive cells conferred by Gcm^R59L^ is due to a differential rate in continuous induction of glial cells due to compromised protein stability. To address this question, Gcm or Gcm^R59L^ expression was induced uniformly in the whole embryo by heat shock at 37 °C using *heat-shock Gal4 (HS-Gal4*) 6 hours after eggs laying (AEL, Supplementary Fig. S4a). After an hour-long induction, embryos were collected at two different time points: early 11–12 hours AEL and late 15–16 hours AEL. Analysis of embryos from these two time points allows us to depict Repo-positive cells in a temporal manner. Interestingly, whereas number of glial cells continued to increase in the later stage upon Gcm overexpression (44.7 ± 1.4 to 61.9 ± 1.8 per hemisegment, Supplementary Fig. S4f,h), the number was not dramatically unregulated compared to that at the earlier time point upon Gcm^R59L^ overexpression (40.7 ± 0.9 to 47.1 ± 2.1 per hemisegment, Fig. S4g,h). A significant difference was detected between Gcm and Gcm^R59L^ overexpression at the later time point (61.9 ± 1.8 vs 47.1 ± 2.1 per hemisegment, p < 0.001, Supplementary Fig. S4h). Yet, glial cells were induced at the early stage to a similar extent either when Gcm or Gcm^R59L^ was overexpressed (44.7 ± 1.4 vs 40.7 ± 0.9, Supplementary Fig. S4c,d). Similar results were obtained when a different individual counts the glial cell number (data not shown). These results suggest that glial cells are properly induced upon Gcm^R59L^ overexpression. Yet, unstable Gcm^R59L^ fails to sustain more glial cell formation, ultimately leading to a lower number of glial cells at later stages.

### PEST domain is required for Gcm protein stability

**Figure 4:**
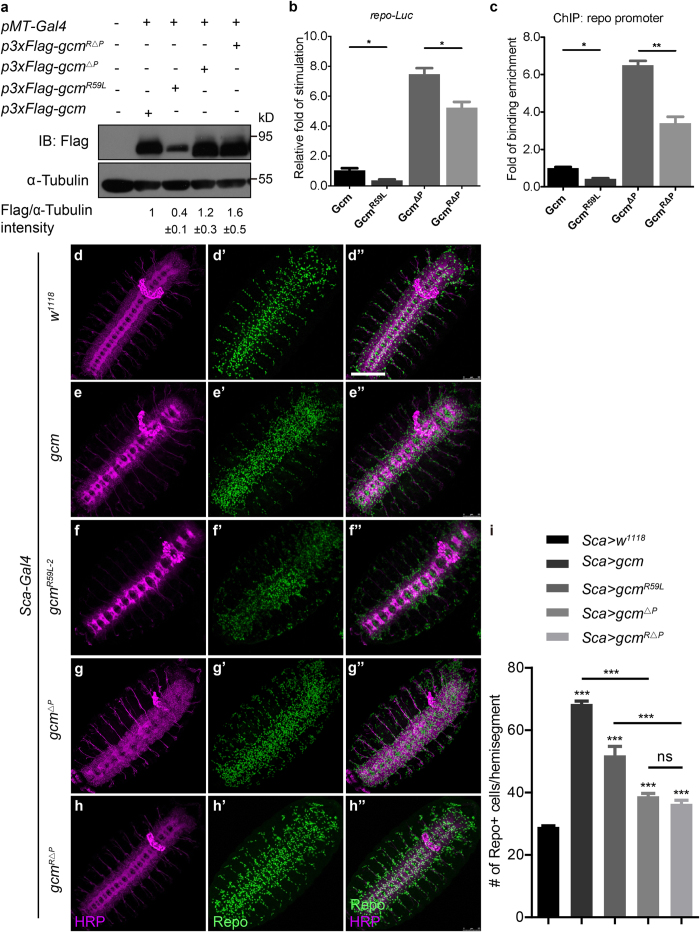
R59 and PEST domain contribute independently to Gcm protein stability. (**a**) Western blot analysis of Gcm, Gcm^R59L^, Gcm^ΔP^, and Gcm^RΔP^ protein levels. Quantifications for relative band intensity of Flag to α-Tubulin were shown at the bottom. Western blot gels have been run under the same experimental conditions. (**b**) Luciferase reporter assays were done for cells transfected with *p3xFlag-gcm, p3xFlag-gcm*^*R59L*^, *p3xFlag-gcm*^*ΔP*^, or *p3xFlag-gcm*^*RΔP*^. Note a significant difference was detected between Gcm^ΔP^ and Gcm^RΔP^. (**c**) ChIP analysis demonstrated that Gcm^ΔP^ and Gcm^RΔP^ bind DNA. ChIP assays were performed using S2 cell lystates tranfected with the above plasmids. Each ChIP experiment was repeated three times (n = 3). (**d**–**h”**) Confocal projections of Stage 15 embryos labeled with HRP (magenta) and Repo (green) for the following genotypes: *Sca-Gal4* > *w*^*1118*^ (**d**–**d”**, control), *Sca-Gal4* > *UAS-gcm* (**e**–**e”**), *Sca-Gal4* > *UAS-gcm*^*R59L-2*^ (**f**–**f”**), and *Sca-Gal4* > *UAS-gcm*^*ΔP*^ (**g**–**g”**), and *Sca-Gal4* > *UAS-gcm*^*RΔP*^ (**h**–**h”**). Scale bar: 100 μm. (**i**) Statistics for the glial cell number per hemisegment were shown for embryos carrying the above genotypes. n = 80 (*w*^*1118*^ control), n = 80 (*UAS-gcm*), n = 35 (*UAS-gcm*^*R59L-2*^), n = 52 (*UAS-gcm*^*ΔP*^), and n = 36 (*UAS-gcm*^*RΔP*^). Note a significant difference between embryos expressing *gcm* or *gcm*^*R59L*^ with or without the PEST domain. Averages are mean ± SEM, * represents p < 0.05, ** represents p < 0.01, and *** represents *p* < 0.001 by Student’s t test between two groups and one-way ANOVA test among multiple groups. ns means no significance.

To further dissect differential requirements for Gcm protein stability, and seek if the N-terminus R59 cooperates with other distinct pathways, we investigate the function of a destabilizing PEST domain carried by Gcm. PEST encodes peptides enriched with proline (P), glutamic acid (E), serine (S), and threonine (T), and has been shown to signal rapid protein turnover^35^. Despite the fact that PEST is a common degradation signal in a variety of proteins, its role in controlling Gcm protein stability has not been investigated. Using site-directed mutagenesis approach to remove the PEST domain, our Western blot analysis showed that Gcm proteins lacking the PEST domain (henceforth Gcm^ΔP^) accumulated (Fig. 4a). Increased protein levels upon PEST deletion also resulted in an increase in Gcm-DNA binding and transcriptional activity as revealed by luciferase reporter and ChIP analyses (Fig. 4b,c). Not only more chromatin DNA was found to associate with Gcm^ΔP^ proteins, a higher level of transcriptional activity was also detected when overexpressing Gcm^ΔP^ in S2 cells. These results suggest that PEST domain is required in controlling Gcm protein stability and Gcm^ΔP^ proteins are functionally active to bind DNA and activate gene transcription.

### N-terminal R59 and PEST domain contribute independently to Gcm protein stability

We then sought to determine if PEST-mediated Gcm protein stability requires R59. A Gcm variant was constructed to contain both R59L mutation and PEST deletion (denoted as Gcm^RΔP^). We found that in the presence of both changes, Gcm^RΔP^ protein levels were higher than Gcm^R59L^, yet similar to Gcm^ΔP^ (Fig. 4a). This piece of evidence suggests that Gcm proteins accumulate upon removal of PEST domain with or without the presence of R59L. However, despite the fact that protein levels were restored, Gcm^RΔP^ proteins did not bind DNA nor activate downstream gene transcription as efficiently as Gcm^ΔP^ (Fig. 4b,c). These results indicate that R59L mutation affects transcriptional activity and DNA binding in the absence of PEST, different from the results in the presence of MG132 (Figs 2f and 3g). Taken together, lacking the PEST domain causes an increase in Gcm protein levels irrespectively of the R59L mutation. Thus, N-terminal R59 and the PEST domain are both crucial yet independently contribute to Gcm protein stability.

Interestingly, analysis of embryos expressing either Gcm^ΔP^ or Gcm^RΔP^ revealed a significant increase in glial cell number, yet not as many as in embryos expressing Gcm or Gcm^R59L^, suggesting that these proteins lacking the PEST domain act less strongly than Gcm or Gcm^R59L^ *in-vivo* (compare Fig. 4e’, and statistics in Fig. 4i). These results are counterintuitive as increasing Gcm protein levels in the absence of PEST domain suggests a higher efficiency in glial cell induction. One possibility is that in addition to the control of protein stability, PEST domain is required for other Gcm functions such as DNA binding or transcription activation as shown for Gcm^RΔP^ (Fig. 4b,c).

### Gcm and Gcm^R59L^ interact with PKC

Above results suggest that R59L mutation causes distorted Gcm protein stability and affects glial cell number independently of the PEST domain. It is then intriguing to investigate the mechanism of how this mutation affects Gcm function. First, taken advantage of previously available structure of the mouse Gcma DNA binding domain, we used Swiss Model to predict the structure of Gcm or Gcm^R59L^ DNA binding domain (Supplementary Fig. S5). Based on these structures and our previous findings on DNA binding and transcriptional activity, replacing R59 to L does not seem to create a dramatic distortion in the structural level that affects its DNA binding ability or transcriptional activity, despite the fact that potential interaction between R59 and other amino acids could have lost.

**Figure 5:**
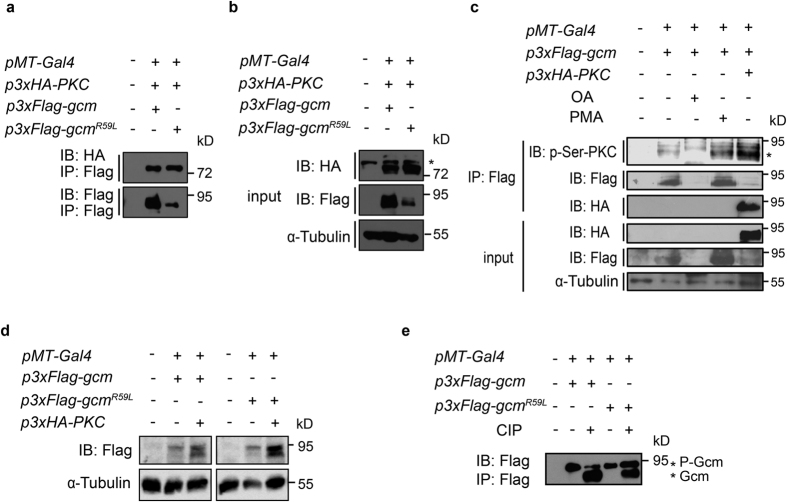
PKC is involved in regulating Gcm and Gcm^R59L^ phosphorylation. (**a**,**b**) Co-IP analysis showed that both Gcm and Gcm^R59L^ interact with PKC, albeit with a different efficiency. Inputs were shown in (**b**). Asterisk on the right side indicated non-specific background bands. (**c**) Gcm expressing S2 cell lysates were treated with OA, PMA, or co-transfected with PKC, pulled down with the anti-Flag antibody, and analyzed with the anti-p-Ser-PKC antibody (top panel), the anti-Flag antibody, (second panel), and the anti-HA antibody (third panel). Inputs for each column were shown in the bottom three panels. Asterisk on the right side indicated 3xFlag-Gcm. Note an increase in the signal intensities when Gcm was co-transfected with PKC or treated with the PKC activator PMA. (**d**) S2 cell lysates transfecting Gcm or Gcm^R59L^ and PKC were analyzed by 10% SDS-PAGE gel. Note the intensities for the upper slower migrating band representing phosphorylated Gcm or Gcm^R59L^ were enhanced upon PKC co-transfection and more phosphorylated Gcm^R59L^ (the upper slower migrating band) were detected compared to Gcm, both in the presence of PKC. (**e**) S2 cell lysates transfected with Gcm or Gcm^R59L^ were pulled down by the anti-Flag antibody, treated with CIP, subsequently analyzed by SDS-PAGE. Note that two bands designated with asterisks represented phosphorylated Gcm (P-Gcm) and dephosphorylated Gcm (Gcm). Gcm^R59L^ exhibited a higher phosphorylation level than Gcm upon CIP treatment. Western blot gels have been run under the same experimental conditions.

We then search for other possible modifications caused by the R59L mutation. Since Gcm^R59L^ interacts with the F-box protein Slimb and the prerequisite for the recognition is Gcm phosphorylation, it is possible that a change in the Gcm phosphorylation status occurs, affecting Gcm^R59L^ interaction with Slimb and its ubiquitination levels. Based on the speculation and results from previous studies that implicated Protein Kinase C (PKC) as a kinase for human Gcma (hGcma) phosphorylation^26^,^27^, we first tested whether Gcm proteins interact with PKC in *Drosophila*. With readily access to tools for one of the six *Drosophila* PKCs, *PKC53E*, we cloned *PKC53E* into the *pUAST* expression vector carrying a N-terminal HA epitope tag in three tandem repeats (3xHA, henceforth denoted as PKC in the text). To analyze interaction between Gcm proteins and PKC, Co-IP analysis was done using S2 cells co-transfected with Gcm or Gcm^R59L^ and PKC. Interestingly, whereas Gcm interacted with PKC, Gcm^R59L^, with a lower protein level, pulled down similar amount of PKC proteins (Fig. 5a,b). These results suggest that both Gcm and Gcm^R59L^ interact with PKC, and Gcm^R59L^ interacts with PKC in a potentially more efficient manner.

### Gcm exhibits a higher level of Serine phosphorylation in the presence of PKC

To further demonstrate that Gcm is a potential PKC substrate, protein lysates from S2 cells transfected with Gcm were analyzed with an antibody against phosphorylated Serine residues on PKC substrates (anti-p-Ser-PKC). These Gcm expressing cells were also treated with okadaic acid (OA, inhibitor of PP1 and PP2A phosphatases), Phorbol-12-Myristate-13-Acetate (PMA, a potent PKC activator), or co-transfected with PKC. Gcm proteins in the pull downs using the anti-Flag antibody were detected with the anti-p-Ser-PKC antibody and indicated with an asterisk on the right side (top panel, Fig. 5c). Interestingly, a higher level of Gcm proteins were detected by the anti-p-Ser-PKC antibody when cells were treated with PMA or co-transfected with PKC, suggesting that Gcm proteins are more phosphorylated on the Serine residues when PKC is activated or in the presence of PKC. These results further support our hypothesis that PKC mediates Gcm phosphorylation.

### A higher level of potentially phosphorylated Gcm^R59L^ was detected in the presence of PKC

Since both Gcm and Gcm^R59L^ interact with PKC, and PKC plays a role in Gcm Serine phosphorylation, we then sought to determine if Gcm^R59L^ protein phosphorylation is affected by PKC. By analyzing the Gcm migrating bands separated to a greater extent, different patterns emerged, and the upper slower Gcm migrating bands in its phosphorylated form appeared to be heavier in intensities in the presence of PKC (Fig. 5d). Strikingly, the intensities of the upper slower Gcm^R59L^ migrating bands were enhanced upon PKC expression, suggesting a higher level of Gcm^R59L^ proteins remain phosphorylated in the presence of PKC (Fig. 5d). On the other hand, treatment with the alkaline phosphatase (CIP) revealed that a higher level of Gcm^R59L^ that potentially represents the phosphorylated form was detected migrating slower on top upon CIP treatment, whereas most of the Gcm migrated faster and potentially represented the dephosphorylated form (the lower Gcm migrating bands, Fig. 5e). These results indicate that Gcm^R59L^, differed from Gcm, exhibits altered phosphorylation likely mediated by PKC.

### Human Gcmb^R47L^ (hGcmb^R47L^) overexpression causes a slight increase in glial cell number similarly as hGcmb

**Figure 6:**
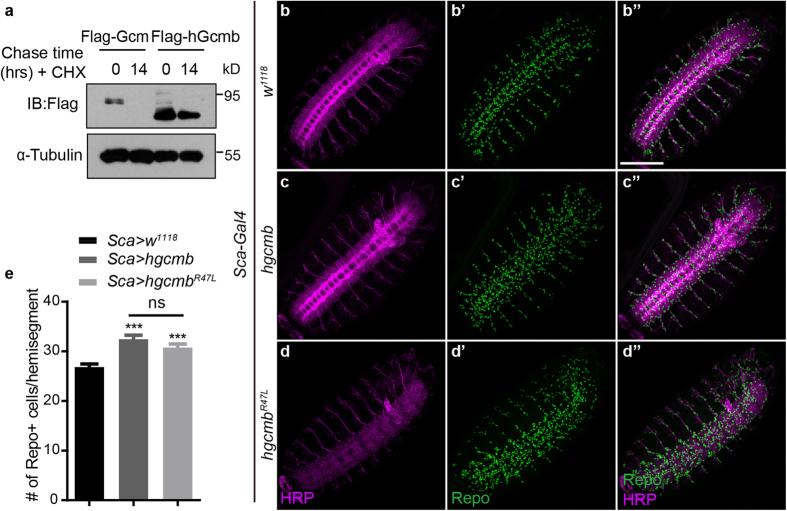
hGcmb^R47L^ induces Repo-positive cells similarly as hGcmb. (**a**) Protein extracts from S2 cells transfected with *gcm* or *hgcmb* were harvested and analyzed in the presence or absence of CHX. Note the persistent protein levels up to 14 hours for hGcmb. Western blot gels have been run under the same experimental conditions. (**b**–**d**) Confocal projections of Stage 15 embryos expressing *hgcmb* and *hgcmb*^*R47L*^ were labeled with HRP (Magenta) and Repo (Green) for the following genotypes: *Sca-Gal4* > *w*^*1118*^ (**b**–**b”**, control), *Sca-Gal4* > *hgcmb* (**c**–**c”**), and *Sca-Gal4* > *hgcmb*^*R47L*^ (**d**–**d”**). Scale bar: 100 μm. (**e**) Statistics for the glial cell number per hemisegment were shown for embryos carrying the above genotypes. n = 40 (*w*^*1118*^ control), n = 51 (*hgcmb*), and n = 40 (*hgcmb*^*R47L*^). Note a significant difference between embryos expressing *hgcmb* or *hgcmb*^*R47L*^ and the control. Averages are mean ± SEM, *represents p < 0.05, **represents p < 0.01, and ***represents *p* < 0.001 by Student’s t test between two groups and one-way ANOVA test among multiple groups. ns means no significance.

Since R59L is a conserved mutation implicated in the disease hypoparathyroidism, and unexpectedly affects *Drosophila* glial cell number, we then wonder if the same mammalian *hgcmb* mutation acts similarly on its protein stability and *Drosophila* glial cell formation. To address this, *hgcmb* and *hgcmb*^*R47L*^ carrying the conserved R to L mutation (R47L) were cloned into the *pUAST* vector for S2 cell expression and transgenic flies analysis. First, hGcmb protein stability was analyzed side by side with *Drosophila* Gcm by CHX pulse chase analysis. Surprisingly, hGcmb protein levels persisted up to 14 hours after CHX addition while *Drosophila* Gcm has completed degraded by then (Figs 1c and 6a). This piece of evidence suggests that hGcmb is more stable and its protein stability is controlled via different mechanisms from *Drosophila* Gcm.

Taken advantage of the newly made transgenic flies, glial cells were analyzed in similar settings as before in embryos overexpressing hGcmb or hGcmb^R47L^ (Fig. 6b–e). Interestingly, the increase in glial cell number upon either hGcmb or hGcmb^R47L^ overexpression was not as obvious as in embryos overexpressing Gcm (compare Fig. 6b’ to Fig. 3b’ and f, hGcmb: 32.4 ± 0.8 to 26.8 ± 0.6 cells per hemisegment, p < 0.001, hGcmb^R47L^: 30.7 ± 0.7 to 26.8 ± 0.6 cells per hemisegment, p < 0.001, Fig. 6e). Furthermore, no significant difference in glial cell number was detected between hGcmb and hGcmb^R47L^, suggesting that *Drosophila* glial cells may not be a perfect system for assaying hGcmb function.

## Discussion

The *gcm* family genes are important transcriptional regulators in a variety of processes including neural stem cell induction, mammalian placenta and parathyroid gland development, and specification and differentiation of the cell type glia in *Drosophila*. By virtue of their importance, multiple and complex mechanisms have been evolved to regulate their functions. Here we demonstrate that a hypoparathyroidism-associated mutation R59L regulates Gcm protein stability via SCF-dependent ubiquitination. Altering the residue does not create deficit in DNA binding, and reporter assay suggests that Gcm^R59L^ is still functional in regulating downstream gene expression. Strikingly, Gcm^R59L^ undergoes hyperubiquitination, triggering the reduction in overall protein levels. Gcm^R59L^ proteins induce glial formation, yet are not competent as wild-type Gcm at later stages of embryo development, consistent with its reduced protein stability. Furthermore, R59L-induced Gcm degradation is independent of the PEST-implicated protein turnover, while both contribute to the regulation of Gcm protein stability. Overall, our work uncovers a regulatory pathway for Gcm protein degradation induced by a disease-related mutation, providing insights on the underlying mechanism of Gcm function.

How does R59L mutation signal SCF complex-mediated degradation? Our results indicate that R59L regulates Gcm protein stability by controlling its ubiquitination levels. Gcm^R59L^ proteins exhibit a strikingly high ubiquitination levels compared to Gcm, suggesting that mutating R59 to L renders Gcm protein more susceptible to ubiquitination (Fig. 2). The strikingly high ubiquitination levels could be due to disruption in interacting with the ubiquitination machineries (i.e. F-box proteins) or a change in the phosphorylation status as phosphorylation is often a prerequisite for ubiquitination^36^,^37^. Our results support the latter hypothesis. First, a roughly similar efficiency was found for Gcm and Gcm^R59L^ proteins to interact with Slimb, suggesting that Gcm^R59L^ is capable of binding to the F-box proteins, and that R59L mutation promotes ubiquitination via a mechanism that does not interfere with F-box protein binding (Fig. 2).

Next, our results indicate that Gcm^R59L^ proteins exhibit altered phosphorylation, suggesting that change in Gcm^R59L^ phosphorylation could be a key to its dysregulated ubiquitination levels (Fig. 5). Furthermore, we speculate that a change in the phosphorylation status for Gcm^R59L^ is due to difference in PKC interaction and the efficiency of PKC-mediated Gcm phosphorylation. PKC interacts with both Gcm and Gcm^R59L^, yet with a greater efficiency to Gcm^R59L^. In addition, increasing Gcm Serine phosphorylation was found when cells were treated with the PKC activator PMA or in the presence of PKC. Altogether, these findings suggest that PKC is a kinase candidate for Gcm phosphorylation and Gcm^R59L^ is potentially a highly accessible form for PKC phosphorylation (Fig. 5). We also show that a higher portion of Gcm^R59L^ proteins remain possibly phosphorylated upon CIP treatment and PKC overexpression, suggesting an involvement of PKC in altering Gcm^R59L^ phosphorylation. Interestingly, previous studies have similarly implicated a role for PKC kinase in mammalian Gcma phosphorylation^26^,^27^. Our results are consistent with the notion that PKC plays a major role in the phosphorylation of Gcm proteins across species. Taken together, R59L mutation tunes Gcm ubiquitination levels, possibly via prior phosphorylation and/or corresponding conformational changes, thus becoming less steady and destabilized.

On the other hand, our results indicate that PEST domain implicated in rapid protein turnover is also required for Gcm protein stability, as depletion of this domain leads to Gcm protein accumulation. However, PEST-mediated Gcm protein stability is independent of R59 as PEST-deleted Gcm proteins (both Gcm^ΔP^ and Gcm^RΔP^) accumulated whether or not R59L mutation is present. Furthermore, increase in Gcm protein levels by PEST deletion does not fully restore R59L-mediated Gcm activity like the MG132 treatment did, suggesting that PEST does not work downstream of R59L, and both may convey independent signals for regulating Gcm protein stability. Therefore, we conclude that the R59L mutation and PEST domain are both crucial yet independent determinants in Gcm protein stability regulation (Fig. 4).

Despite the fact that overexpression of either Gcm or Gcm^R59L^ induces glial formation, Gcm^R59L^ acts in a less competent manner as the number of differentiated glia is significantly less compared to Gcm overexpression (Fig. 3 and Supplementary Fig. S3). Furthermore, Gcm^R59L^ overexpression fails to increase glial cells at a later stage (Supplementary Fig. S4). It is possible that insufficient Gcm^R59L^ proteins are available for activating downstream gene expression such as Repo, even though Gcm^R59L^ proteins bind to DNA and activate gene expression with a similar efficiency as the wild-type Gcm. Thus, initial conversion from precursor cells to glia is successful in the context of Gcm^R59L^ possibly due to a lower threshold of protein requirement. At a later stage, however, the need for Gcm activity to further increase glial cells, to maintain the glial differentiation, or both, increases above the availability of Gcm^R59L^ protein pool, thus leading to fewer glial cell number compared to wild-type Gcm.

During the initial assessment of possible intrinsic factors contributing to Gcm protein stability, R59L was originally identified as a conserved mutation detecting in the *hgcmb* gene in hypoparathyroidism patients (*hgcmb*^*R47L*^). Interestingly, hGcmb^R47L^ proteins are stable, yet defective in DNA binding and transcriptional activation^19^,^32^,^33^,^34^. These results are in contrast to the ones reported in our present study and raise concerns on the difference in species and homologous protein function. First, previous studies have reported that the conserved R47L mutation in hGcmb does not cause distorted protein stability, suggesting that our observation on reduced Gcm^R59L^ protein stability is not a universal consequence caused by R59L structural distortion (Supplementary Fig. S5). Next, hGcmb proteins exhibit a much longer half-life compared to the *Drosophila* Gcm (up to 14 hours after the CHX chase, Fig. 6a), reflecting the difference in degradation mechanism of hGcmb vs. Gcm protein stability. Furthermore, given that human Gcm proteins regulate placenta and hypoparathyroid development^20^,^38^,^39^ and there is no direct evidence supporting that human Gcm proteins are involved in glial formation and differentiation, it is noteworthy to mention that *hgcmb* and *hgcmb*^*R47L*^ induce glial cells only vary slightly in our system (Fig. 6b–e). We were unable to detect a major difference between hGcmb and hGcmb^R47L^ when analyzing their overexpression effect in glial cell formation, as the increase in glial cell number is relatively small in either scenario. Thus, comparison between the half-life of hGcmb and hGcmb^R47L^ will be less meaningful in this case.

In this study, we identify a distinct regulatory signal in controlling *Drosophila* Gcm protein stability conferred by the hypoparathyroidism-related mutation. This regulatory mechanism helps to elucidate further how the protein stability of a transcription factor is controlled by the ubiquitination-mediated degradation, thus providing potential implications on the mechanism of *in-vivo* cell fate determination and differentiation.

## Materials and Methods

### Fly Strains

Flies were maintained at 25 °C on normal food. All strains were obtained from Bloomington Stock Center, VDRC, or as gifts from colleagues (*UAS-gcm* fly, Angela Giangrande). All fly crosses were carried out at 25 °C with standard laboratory conditions unless noted otherwise. All transgenic flies including *hgcmb, hgcmb*^*R47L*^, *gcm*^*ΔP*^, and *gcm*^*RΔP*^ flies were created by embryonic microinjection performed in the *Drosophila* Technique Platform of Institute of Biochemistry and Cell Biology (SIBS, CAS, China), except the *gcm*^*R59L*^ flies microinjected by Chiu-Yang Tang, Dr. Henry Sun, Institute of Molecular Biology, Academia Sinica.

### Cloning and Cell-based Studies

To generate above transgenic flies, *hgcmb* and *hgcmb*^*R47L*^ plasmids, generous gifts from R.V. Thakker, were subcloned using standard protocols. Primer sequences designed for PCR are: 5′-CCGGCGGCCGCGGTGCAG-3′ and 5′-TCAAAAATCCTCATTGTTGTAGGTAAAG-3′. Purified PCR products were then cloned into the *pUAST-3xFlag* Gateway vector (Invitrogen). Site-directed mutagenesis using PCR was done to create *gcm*^*R59L*^, *gcm*^*ΔP*^, and *gcm*^*RΔP*^ plasmids.

*Drosophila* S2 cells were cultured in Schneider’s *Drosophila* medium (Gibco) in 6-well plates or 100 mm dishes. S2 cell transfection was performed with cells at confluent density and using 0.2 μg of *MT-Gal4* and each *UAS* construct for 6-well plates or 0.5 μg of *MT-Gal4* and each *UAS* construct for 100 mm dishes. Cells were treated with CuSO_4_ (1 mM, Sigma C8027) 24 hours post transfection, and harvested at 48 hours post transfection. For CHX pulse chase experiment, transfected S2 cells were treated with CHX (10 μM, Sigma C7698) and harvested at different time points including 0, 3, 5, and 8 hours after CHX addition. Cells were lysed in lysis buffer (1% NP-40, 20 mM Tris-HCl pH 8.0, 150 mM NaCl, 2 mM EDTA, 1 mM PMSF, proteinase inhibitor cocktail, Roche) and analyzed by SDS-PAGE. For RT-PCR, total RNAs were extracted from S2 cells with Trizol and reversely transcribed into cDNA using the QuantScript RT Kit (Tiangen Biotech). Primers sequences are as follows: endogenous *gcm* 5′ primer 5′-GTTTTGAACGGCATGCCTATAACAATGCCAGTACC-3′, *3xFlag gcm* 5′ *primer* 5′-ATGGACTACAAAGACGACGACGACAAAG-3′ and *gcm* 3′ primer 5′-CTAGCAATAGATGGGATCCGTGCTG-3′.

### Western blot and Immunoprecipitation

For Western blot, protein samples were extracted from transfected S2 cells and analyzed by standard biochemical procedures. Primary antibodies used include: anti-α-tubulin (mouse, Sigma B5–1–2, 1:500000), anti-p-Ser-PKC (rabbit, Cell Signaling #2261 S, 1:300), anti-Flag-HRP (rabbit, Sigma A8592, 1:1000), anti-HA-HRP (rat, Roche 3F10, 1:1000), or anti-c-Myc-HRP (mouse, Santa Cruz Biotechnology sc-40 HRP, 1:500). For Co-IP analysis, protein lysates from S2 cells were harvested and lysed using the standard protocols. Flag antibody-conjugated beads (mouse, sigma A2220) or the anti-HA affinity matrix (rat, Roche 11815016001) were used to pull down the corresponding proteins for 2–2.5 hours at 4 °C. After serial washes with lysis buffer, resins were subjected to SDS-PAGE for analysis with antibodies described above. For experiments with drug treatments, drugs used were: MG132 (50 μM, BioVision), E64 (1 μM, Merck Millipore), OA (0.1 μM, Sigma 75320), and PMA (0.01 μM, Sigma P8139).

For the CIP dephosphorylation assay, transfected cells were lysed in lysis buffer (1% NP-40, 20 mM Tris, pH 8.0, 150 mM NaCl, 2 mM EDTA, 14 mM β-mercaptoethanol, protease inhibitor, and 1 mM PMSF). Supernatants from cell lysates were incubated with or without CIP (New England Biolabs) at 37 °C for 40 min and analyzed by Western blot.

### Immunohistochemistry and Microscopy

Stage 12 (7–9 hours) and 15 embryos (11–13 hours) collected from Grape plates were dissected and fixed with 4% formaldehyde in 1X PBS following standard protocols previously described. Reagents used include: Normal Donkey Serum (0.5%, NDS, Jackson Immunoresearch), anti-HRP-TRITC (Jackson Immunoresearch, 1:500), mouse anti-Repo (DSHB, 1:100). All other secondary antibodies were purchased from Jackson Immunoresearch. Images were captured with Leica TCS SP5 confocal microscopy.

### Statistical Analysis

For Western blot images, experiments were repeated at least three times independently and band intensities were measured and quantified using Image J software. Quantification ratios were indicated for the particular image shown in figures. For embryos, number of glial cells was counted according to previous protocols^27^ for regions from the midline (yellow arrow) to the five lateral chordotonal (LCH) organ-associated glia (yellow arrowheads). To avoid any unconscious bias on cell count, a second person was asked to count the glial cell number. More than 40 hemisegments (A1–A4 from average 10 embryos for each genotype) were chosen and quantified. Significance (indicated with asterisks, *p < 0.05, **p < 0.01, ***p < 0.001) and P-values were calculated using unpaired two-tailed T-test between two groups and one-way ANOVA with Bonferroni multiple comparison test among three groups or above. ns means no significance.

### ChIP and real-time PCR analysis

ChIP assay was performed as described previously^40^. Crosslinked, sonicated chromatin was precleared before being incubated with 2.5 μg of the indicated antibodies and rotated at 4 °C overnight. Normal mouse or rabbit IgG (Millipore) was used for the mock immunoprecipitation. After extensive washes, immunocomplexes were treated with Proteinase K and decrosslinked. Bound DNA in the precipitates, as well as input DNA (1/10 fragmented chromatin), was extracted, purified, and subjected to real-time PCR analysis using primers corresponding to a region of the *repo* promoter (−1,337 to −1,138; forward primer, 5′-ATCTGGCCAAAAGGTCACAC-3′; reverse primer, 5′-CTCATGAGGGTGGTTCCATT-3′). Real-time PCRs were conducted on the Bio-Rad iQ5 Gradient Real-Time PCR system, using the 2 × SYBR-Green Master mix (Bio-Rad, USA). Results were corrected for non-specific binding to IgG (whose values were considered as 1 and not shown in the bar graphs). Triplicate PCRs for each sample were carried out.

### Promoter reporter constructs and Luciferase reporter assay

Luciferase reporter construct containing a 2.2-kbp DNA fragment corresponding to the *repo* upstream regulatory region (−100 to −2,300) was generated. This region was previously shown to confer the expression and activity of *repo in vivo*, and is known to harbor multiple Gcm binding sites (GBS)^41^. The corresponding fragment was amplified by PCR (forward primer, 5′-CCTTGAAGCCAGACCCACATAATTGG-3′; reverse primer, 5′-ACTATCGCCGTGCGAGCGGGACG-3′), purified, and subsequently subcloned into the luciferase vector pGL3-control (Promega). For promoter reporter assay, S2 cells were seeded in six-well plates before being co-transfected (at 50% confluence) with the *repo* promoter reporter constructs and expression vectors using Effectene (Qiagen). After 2-day incubation, cells were lysed in Reporter Lysis 5 × Buffer (Promega) with two rounds of freeze thaw, followed by incubation at 4 °C. Cell debris was removed by centrifugation, and luciferase activity in the supernatant was measured by a Dual-Luciferase Reporter Assay System (Promega). The relative light units were firefly luciferase units normalized to absorption units of the co-expressed β-gal.

## Additional Information

**How to cite this article**: Xi, X. *et al*. The hypoparathyroidism-associated mutation in *Drosophila* Gcm compromises protein stability and glial cell formation. *Sci. Rep.* **7**, 39856; doi: 10.1038/srep39856 (2017).

**Publisher's note:** Springer Nature remains neutral with regard to jurisdictional claims in published maps and institutional affiliations.

## Supplementary Material

Supplementary Information
