# Supplementary material for: The hypoparathyroidism-associated mutation in Drosophila Gcm compromises protein stability and glial cell formation
Source: Sci Rep. 2017 Jan 4;7:39856. doi: 10.1038/srep39856 (PMC5209662; doi:10.1038/srep39856)
Supplement: Supplementary Information [file srep39856-s1.pdf]

Supplementary Information

The hypoparathyroidism-associated mutation in *Drosophila* Gcm compromises protein stability and glial cell formation

Xiao Xi[1, 2, 3], Lu Lu[1, 2, 3], Chun-Chun Zhuge[1, 2, 3], Xuebing Chen[1, 2, 3], Yuanfen Zhai[1, 2, 3], Jingjing Cheng[1, 2, 3], Haian Mao[1, 2, 3], Chang-Ching Yang[4], Bertrand Chin-Ming Tan[4], Yi-Nan Lee[5], Cheng-Ting Chien[5], and Margaret S. Ho[1, 2, 3, ‡]

a

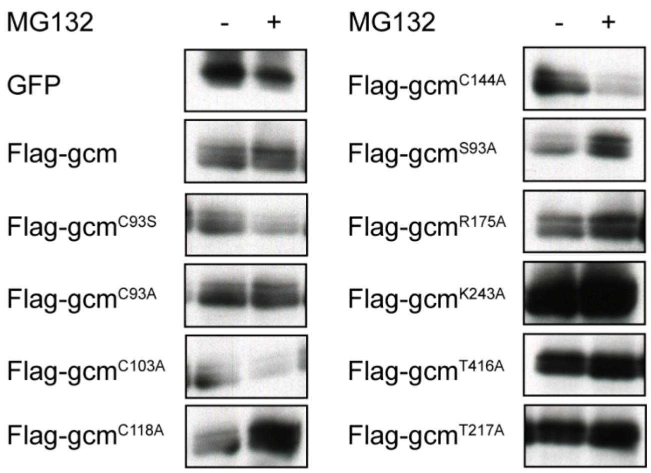

Supplementary Figure S1. Protein levels of Gcm carrying different point mutations. (**a**) Western blots showing expression of different Gcm mutant variants in the absence or in the presence of MG132 (50 μM). Lysates were collected 4 h after MG132 treatment.

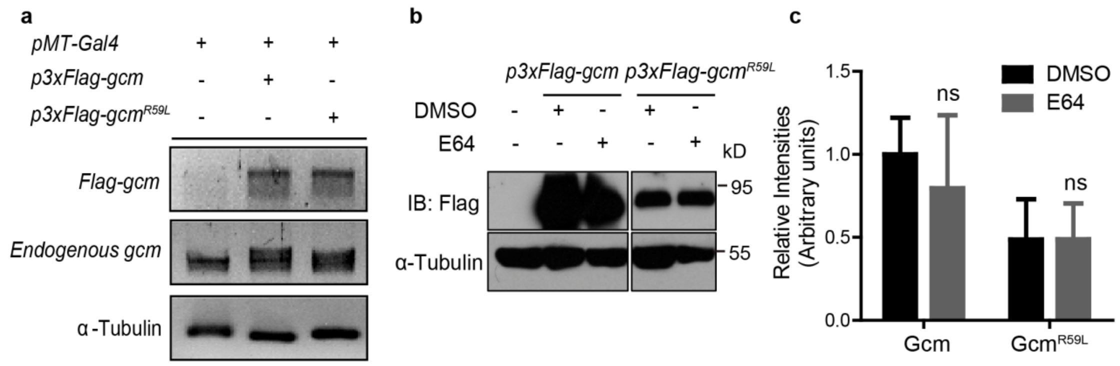

Supplementary Figure S2. Analysis of Gcm^{R59L} transcript by RT-PCR and Gcm^{R59L} protein stability is independent of the lysosome degradation pathway.

(**a**) Total RNAs extracted from S2 cells that expressed endogenous Gcm, exogenous Flag-Gcm and Flag-Gcm^{R59L} were analyzed by RT-PCR for their transcript levels. Control: α-tubulin. There is no difference in the levels of either endogenous or exogenous *gcm* or *gcm^{R59L}* mRNA transcripts. (**b**) Western blots showing expression of Gcm and Gcm^{R59L} in S2 cells in the absence or in the presence of E64 (50 μM). Lysates were collected at 5 h after E64 treatment. (**c**) Quantifications for relative band intensities of Flag to α-tubulin from 3 independent experiments (n=3). Relative band intensities of Gcm or Gcm^{R59L} with or without E64 treatment exhibit no significance in statistics. Western blot gels have been run under the same experimental conditions. Data were shown as mean ± SEM, * represents p<0.05, ** represents p<0.01, and *** represents *p*<0.001 by Student's t test. ns means no significance.

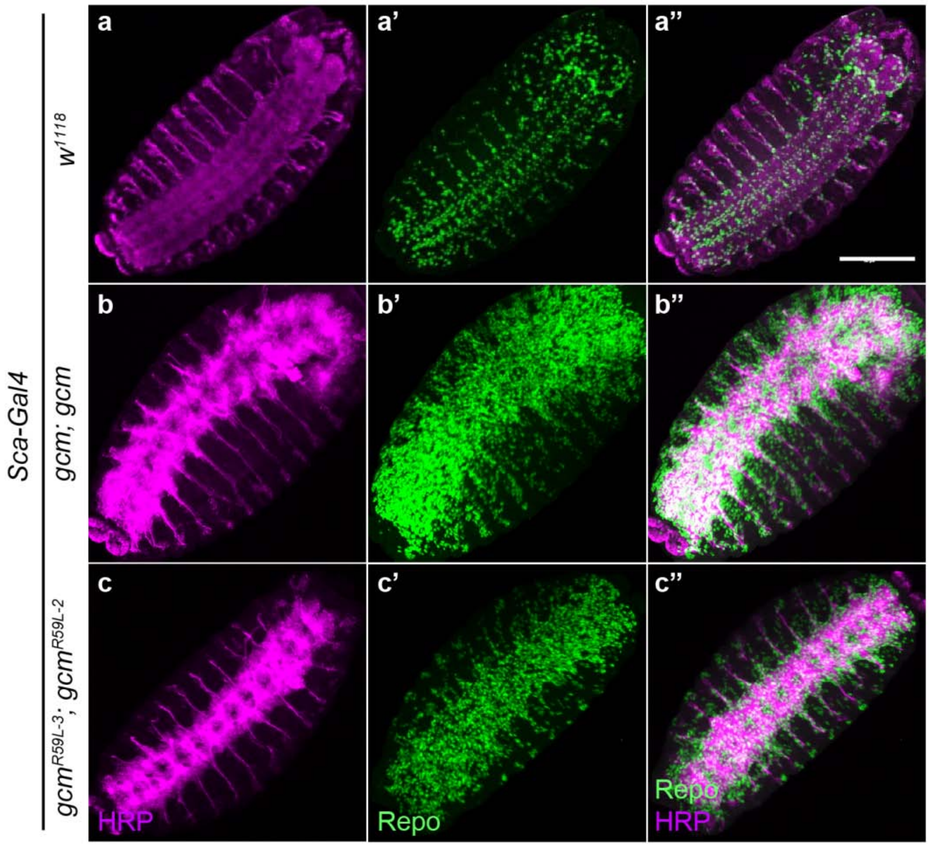

Supplementary Figure S3. Overexpression of Gcm and Gcm^{R59L} by two copies of transgenes. (**a**-**c”**) Confocal projections of Stage 15 embryos were immunostained for HRP (purple) and Repo (green) for the following genotypes: *Sca-Gal4>w^{1118}* (**a**-**a''**), *Sca-Gal4>UAS-gcm; UAS-gcm* (**b**-**b''**), *Sca-Gal4>UAS-gcm^{R59L-3}; UAS-gcm^{R59L-2}* (**c**-**c''**). Scale bar: 100 µm. Note an overall difference in glial cell number (green) between two copies of *gcm* and *gcm^{R59L}*.

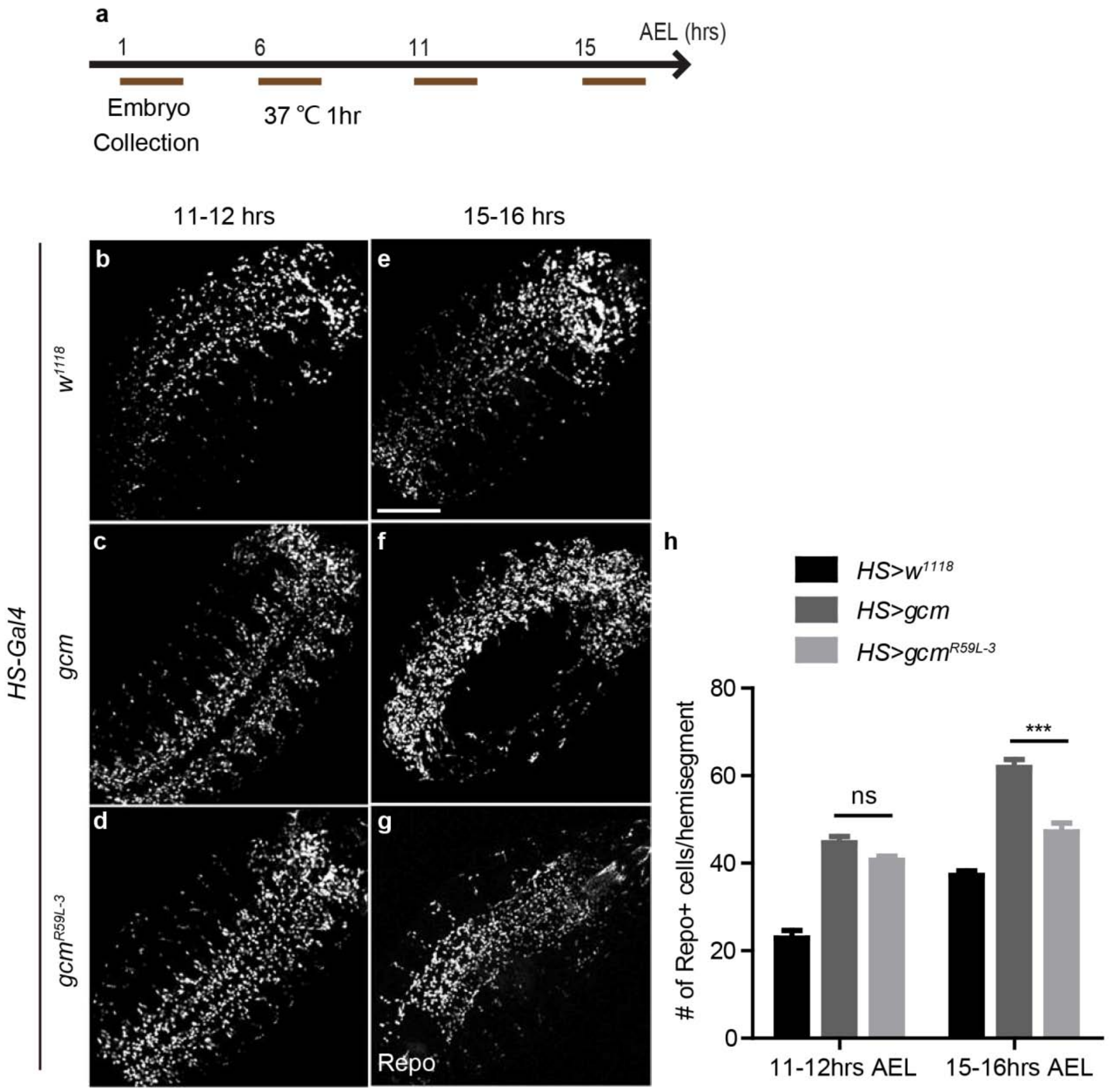

Supplementary Figure S4. Gcm^{R59L} overexpression fails to maintain glial differentiation.

(**a**) A schematic diagram to reveal the timeline for heat shock analysis. Gcm or Gcm^{R59L} overexpression by *heat shock Gal4* (*HS-Gal4*) was induced at 6-7 hours after eggs laying (AEL) by heat shock at 37°C. Each brown line represents an hour long. Collected embryos were analyzed at two different time points: early 11-12 hours AEL and late 15-16 hours AEL. (**b**-**g**) Confocal projections of embryos labeled with Repo (white) for glia with the following genotypes: *HS-Gal4>w^{1118}* (control, **b** and **e**), *HS-Gal4>UAS-gcm* (**c** and **f**),

HS-Gal4>UAS-gcm$^{R59L\text{-}3}$ (**d** and **g**). Scale bar: 100 μm. Note that the glial cell number increased between two time points upon *gcm* overexpression, yet remain similar when *gcm*^{R59L} was overexpressed. (**h**) Numbers of glia per hemisegment were shown for embryos carrying the above genotypes at two time points. For 11-12 hours AEL, n=18 (*w*^{1118} control), n=52 (*UAS-gcm*), and n=59 (*UAS-gcm*$^{R59L\text{-}3}$). For 15-16 hours AEL: n=19 (*w*^{1118} control), n=47 (*UAS-gcm*), and n=23 (*UAS-gcm*$^{R59L\text{-}3}$). Note a significant difference in glial cell number was detected at 15-16 hours AEL between embryos expressing *gcm* and *gcm*^{R59L}. Data were shown as mean ± SEM, * represents $p<0.05$, ** represents $p<0.01$, and *** represents $p<0.001$ by Student's t test between two groups and one-way ANOVA test among multiple groups. ns means no significance.

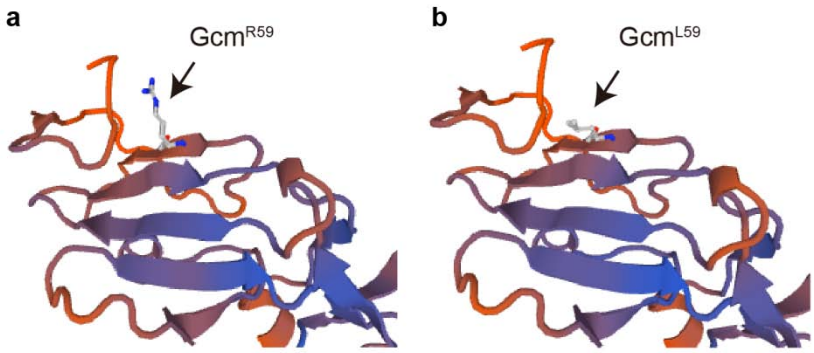

Supplementary Figure S5. Predicted structure of Gcm and Gcm^{R59L} DNA binding domain by Swiss Model.

(**a** and **b**) Based on the published structure for mGcma DNA binding domain, we modeled the structure for Gcm and Gcm^{R59L} using Swiss Model. Black arrows indicated the R59 or L59 residue. β-sheets were shown in blue. Only partial DNA binding domains were shown. Note an overall similarity in the two predicted structures.
